# Shared and unique interoceptive deficits in high alexithymia and neuroticism

**DOI:** 10.1371/journal.pone.0273922

**Published:** 2022-08-31

**Authors:** Giulia Gaggero, Sara Dellantonio, Luigi Pastore, Kelly H. L. Sng, Gianluca Esposito

**Affiliations:** 1 Department of Psychology and Cognitive Sciences, University of Trento, Rovereto, Italy; 2 Department of Education, Psychology, Communication, University of Bari, Bari, Italy; 3 Psychology Program, School of Social Sciences, Nanyang Technological University, Singapore, Singapore; Polytechnic Institute of Coimbra: Instituto Politecnico de Coimbra, PORTUGAL

## Abstract

Interoception is the perception of internal bodily signals. It is considered fundamental to developing emotional awareness. For this reason, interoceptive deficits are often associated with alexithymia, a condition characterized by difficulty identifying feelings (DIF), difficulty describing feelings (DDF), and an externally-oriented style of thinking (EOT). Yet, the atypical interoception found in alexithymia might be of a similar type and/or more serious than those found in other partially overlapping constructs that entail emotional difficulties and behavioural patterns associated with specific emotional styles. Our study explores this issue by examining the relationship between the interoceptive deficits associated with alexithymia and the Big Five personality traits. A non-clinical sample (N = 504) completed the Toronto Alexithymia Scale, the Big Five Inventory and the Multidimensional Assessment of Interoceptive Awareness. Data were analysed using a network analytic approach that conceives psychological traits as networks of interacting symptoms. The estimated network highlighted that EOT is the alexithymia component least associated with interoception and most associated with lower Openness to Experience. Conversely, DIF and Neuroticism are, respectively, the dimensions of alexithymia and the Big Five most highly associated with interoception. We also compared interoceptive abilities in the four groups of participants whose scores were a) high for both alexithymia and neuroticism, b) high only for alexithymia c), high only for neuroticism, and d) low for both. High alexithymia was especially associated with the tendency to ignore sensations of pain or discomfort, while neuroticism was more indicative of the tendency to worry about these sensations. These results suggest that while high alexithymia and neuroticism share some interoceptive deficits, others are unique to alexithymia and contribute to overall lower interoceptive ability in this condition. Our findings suggest that interventions to enhance awareness of bodily sensations can be beneficial especially for profiles who present high neuroticism and alexithymia.

## Introduction

Introduced more than a century ago by Sherrington [1], the term interoception currently refers to the “multisensory, multimodal, integrated percept of the state of the body” [2]. In the literature, we often find the expressions “interoceptive awareness” or, sometimes, “bodily awareness” [3] used to indicate our capacity to consciously perceive and report ongoing sensations from inside the body [4]. Although interoception originally denoted only sensations from the visceral organs (e.g., heartbeat, respiratory rate, sensations from the stomach and the intestines), today its meaning is more inclusive [2] and applies to the overall state of the body, including sensations of temperature or those generated by the musculoskeletal system [5]. The most widely used model for operationalizing interoceptive ability includes three dimensions: i) Interoceptive Sensibility (IS): self-reported interoceptive ability as measured by questionnaires; ii) Interoceptive Accuracy (IAcc): objective performance on interoceptive tasks such as the heartbeat counting task [6]; and iii) Interoceptive Awareness (IAw): metacognitive awareness of interoceptive accuracy measured as the concordance between a subject’s objective interoceptive accuracy and their confidence ratings [7].

Despite constant refinement in the operationalisation of this concept, it is well-established that interoception underpins numerous abilities. One of these is to interpret certain bodily arousal signals as emotional experiences [8–10]. In fact, some authors suggest that individual differences in emotional “style” may reflect differences in sensitivity to interoceptive signals [10]. Moreover, altered interoception might lead to specific deficits in emotion perception and thus also to the development of psychopathologies related to lacking or having a modified perception of own emotions [11]. Given the fundamental role of interoception in emotion processing, it is not surprising that a broad stream of alexithymia research focuses on its relationship to interoception [12, 13].

Alexithymia is a psychological construct consisting of a deficit in the cognitive processing of emotions [14]. Its main features comprise i) difficulties identifying feelings and distinguishing them from sensations of emotional arousal (DIF), difficulties describing feelings (DDF), externally-oriented thinking, and poor imaginal capacity (EOT; [15]). Alexithymia is particularly useful for predicting a variety of clinical conditions which are also linked to low interoceptive awareness, such as psychosomatic disorders, substance-related and addictive disorders, eating disorders [14, 16], and autism spectrum disorders [17–19]. People who score high for alexithymia have difficulties in regulating their emotions [14] as well as in recognizing other people’s emotions or understanding social situations [20–25]. Moreover, they experience predominantly negative emotions [26–28].

There is evidence that high alexithymic traits also occur in the non-clinical population; an incidence between 9 and 13% has been reported in Western countries [29, 30]. In non-clinical populations, alexithymic traits were found to be associated with lowered self-reported interoceptive accuracy [12, 31] and interoceptive awareness [32].

As the description offered above already suggests, recent evidence increasingly supports the claim that alexithymia is accompanied by a lower ability to perceive and interpret interoceptive signals. However, the degree of this association is still debated. Some researchers characterize alexithymia as a general failure of interoception [33], while others adopt a more cautious position [31, 34]. The latter position is largely based on methodological considerations, especially uncertainties concerning how to best measure interoception across different dimensions (i.e. IS, IAcc, IAw). New behavioural tasks for measuring Interoceptive Accuracy (IAcc) are urgently needed given that the widely used Heartbeat Counting Task (HCT) sometimes produces contradictory findings which, overall, do not support the association between IAcc and alexithymia [12, 35]. Despite such concerns, the negative association between alexithymia and self-reported interoceptive ability (IS) is statistically well supported [12, 31].

Yet, there are at least two theoretical issues that also suggest caution in inferring a strong relationship between alexithymia and interoception. Firstly, while atypical interoceptive awareness is certainly a characteristic of alexithymia, it might be possible that not all aspects of this condition are directly related to it [31, 34]. For example, two of the three subscales of the TAS-20 are quite closely related to atypical interoception (DIF and DDF), while the third (EOT) may instead reveal disrupted cognitive processing of the emotional signal. Secondly, the interoceptive deficits found in alexithymia might or might not be of a similar type and/or more serious than those found in other partially overlapping constructs that involve emotional dysregulation, negative affectivity, poor social skills, empathy, and other behavioral patterns related to specific emotional styles such as those captured by the Five-Factor Model of personality (FFM).

This study aims to address these two issues by examining the mutual relationship between each component of alexithymia, self-reported interoception, and the FFM.

The Five-Factor Model of personality (FFM), also known as the Big Five model [36] was proposed over 40 years ago. It aimed to reduce the number of specific personality constructs and provide a shared taxonomy. While researchers may apply different terms and ascribe different definitions to the five personality traits [37], the FFM broadly encompasses extraversion, agreeableness, conscientiousness, openness to experience, and neuroticism. Extraversion represents the tendency to be sociable [38], to engage with the external world, and experience positive emotions [39]. Agreeableness refers to the tendency to be warm [40], kind, and compliant [41], and in so doing, form friendly relationships [42] and promote belongingness-related behaviours [43]. Conscientiousness indicates a tendency for self-discipline [44], and orderly behaviours [45]. Openness to experience refers to a tendency towards intellectual pursuits [46], as well as a preference for the new and unconventional [47]. Neuroticism refers to the tendency to experience negative emotions [38], such as anger, anxiety, stress, guilt, as well as relationship insecurities [48]. These broad dimensions emerged from a factor analysis of questionnaires and adjective checklists, capturing subjects’ dispositions [49, 50]. The Big Five model, consequently, emerged as a descriptive rather than a theoretically driven model of personality. Its strength lies in high psychometric validity [51–53] and replicability in different countries [54].

The idea that the application of the Big Five model would be beneficial to predicting individual well-being is not new. For instance, more than 30 years ago, Costa and McCrae [49] suggested that the Big Five dimensions could replace many commonly used constructs in the psychosomatic field, including that of alexithymia. Specifically, they argued that alexithymia could be included in the openness to experience dimension, because individuals with high alexithymia exhibit reduced openness to experience (individuals with high openness to experience are usually described as inquisitive, open-minded, sensitive to fantasy, feelings, and aesthetic experiences). Their prediction was challenged by results in the empirical literature [55–58], which indicated that the relationship between alexithymia and the Big Five dimensions was more complex than previously assumed [59]. These considerations were highlighted by a recent meta-analysis [60], which shows that alexithymia is positively correlated with neuroticism and negatively correlated with all the other Big Five dimensions, with age moderating the strength of these relationships. Today, neuroticism is probably the FFM dimension that is most studied in connection with well-being, also because it was originally considered to reflect heightened autonomic reactivity [61]. For instance, a few studies [62–64] have suggested that its interaction with alexithymia might play a role in the occurrence of psychosomatic symptoms and subjective health complaints. Moreover, a few studies [65, 66] have already reported that neuroticism is negatively associated with self-reported interoceptive ability.

However, so far, no studies have examined the mutual relationship that neuroticism and the other Big Five dimensions entertain with alexithymia and interoception. In this study, we aim to apply a psychometric network analysis [67, 68] to further clarify which components of both alexithymia and the Five-Factor Model of personality (FFM) are most closely related to self-reported interoceptive ability. Moreover, we will investigate if the interoceptive deficits found in people with high alexithymia differ from those observed in people with high neuroticism and if the co-occurrence of high alexithymia and high neuroticism is associated with general lower interoceptive ability.

Overall, the expected findings should have both theoretical and practical utility, providing insight into the way in which interoception and alexithymia overlap, and into the similarity of interoceptive deficits across different conditions that entail dysregulated emotional ability.

## Materials and methods

The data collection for the current study was part of a broader data collection, involving additional psychological tests. The findings related to the other parts of the project are reported in a separate paper [31]. The protocols of this project were approved by the Nanyang Technological University of Singapore (PSY-IRB 2019–030; IRB 2020-10-016). Participants recruited within the project protocol PSY-IRB 2019–030 signed a written informed consent. Participants recruited within the project protocol IRB 2020-10-016 provided their consent online (they also had the possibility to withdraw their consent after reading the debriefing).

### Participants

Overall, 504 subjects took part in this study. Two groups of participants were separately recruited: 1) a group of English-speaking US residents recruited through Amazon’s Mechanical Turk who received a monetary reward (6$) for completing the survey, and 2) a group of Singaporean university students, who received university credits for their participation. The US group comprised 257 participants (123 female, 134 male; M_age_ = 29.51, SD_age_ = 5.02, age-range = 22–58; 68% White Caucasian; 12% African American; 11% Asian / Pacific Islander; 5% Hispanic American; 4% Multiple Ethnicity/Other). The Singaporean group comprised 247 participants (155 female, 92 male; M_age_ = 21.82, SD_age_ = 2.07, age-range = 18–28; 82% Chinese, 6% Indian, 5% Malay, 2% Eurasian, 5% Other). The collection of groups from different countries and ethnicities was meant to guarantee the generalizability of our results. We conducted preliminary analyses separately for gender and nationality groups. Then, we provided a justification for the combined treatment of data in the main analytical section.

### Measures

The *Big Five Inventory; BFI* [52] consists of 44 items, measuring neuroticism (e.g. “I see myself as someone who can be tense”), extraversion (e.g. “[…] who is talkative”), openness to experience (e.g. “[…] who is curious about many different things”), agreeableness (e.g. “[…] who is helpful and unselfish with others”), and conscientiousness (e.g. “[…] who makes plans and follows through with them”). Responses are rated on a 5-point Likert scale, ranging from 1 “disagree strongly” to 5 “agree strongly”. 16 items are reverse scored. In our sample, Cronbach alphas were as follows: Extraversion = .87; Agreeableness = .78; Conscientiousness = .83; Neuroticism = .87, Openness to Experience = .80.

The *20-item Toronto-Alexithymia Scale; TAS-20* [69] measures three dimensions of alexithymia: Difficulty Identifying Feelings (DIF), e.g., “I am often confused about what emotion I am feeling”; Difficulty Describing Feelings (DDF), e.g., “It is difficult for me to find the right words for my feelings”; Externally Oriented Thinking (EOT), e.g., “I prefer talking to people about their daily activities rather than their feelings”. Total scores range from 20 to 100, with higher scores suggesting higher levels of alexithymia. In our sample, Cronbach alphas were as follows: TAS-20 = .87; DIF = .88; DDF = .81; EOT = .54.

The *37-item Multidimensional Assessment of Interoceptive Awareness; MAIA-2* [70] consists of 8 subscales that reflect dimensions of body awareness: 1) Noticing (e.g. “When I am tense I notice where the tension is located in my body”); 2) Not-Distracting (e.g. “I ignore physical tension or discomfort until they become more severe” reverse-scored); 3) Not-Worrying (e.g. “I can notice an unpleasant body sensation without worrying about it”); 4) Attention Regulation (e.g. “I can pay attention to my breath without being distracted by things happening around me”); 5) Emotional Awareness (e.g., “I notice how my body changes when I am angry”); 6) Self-Regulation (e.g. “When I feel overwhelmed I can find a calm place inside”); 7) Body Listening (e.g. “I listen for information from my body about my emotional state); 8) Trusting (e.g. “I am at home in my body”). Participants judge how often each item applies to them in daily life on a 5-point Likert-type scale ranging from 0 “never” to 5 “always”. Scores on each subscale are the average of corresponding items. Moreover, a total score was computed, from the sum of scores at each subscale. In our sample the subscales’ Cronbach alphas were as follows: Noticing = .64; Not-Distracting = .85; Not-Worrying = .75; Attention Regulation = .86, Emotional Awareness = .80; Self-Regulation = .83; Body Listening = .86; Body Trusting: 0.88.

### Data analysis plan

The Statistical Package R version 3.6.2 for Windows was employed for analysing data.

### Network estimation

Applying the psychological network approach [67, 68], we estimated a weighted partial correlation network, whose nodes were subscores at TAS-20, BFI and MAIA-2. Partial correlation networks offer a better fit with personality and psychological cross-sectional data, where it is important to estimate the magnitude and sign of edges (links between variables, which in our case were construct facets), while edge direction is hard to define given the cross-sectional nature of data [71]. Graphical Least Absolute Shrinkage and Selection Operator (LASSO) [72] in combination with Extended Bayesian Information Criteria (EBIC) model selection [73] were used to obtain a parsimonious representation of connections within the network. The importance of each node in the network was investigated by examining the “strength” centrality metrics, which reveals the degree to which a node is connected with all others by summing up the absolute values of its edge weights. The R *qgraph* (version 1.6.9., [74]) and *bootnet* packages (1.4.3., [75]) were used for these analyses.

### Groups comparisons

Using the clinical cut-off for alexithymia (> = 61), we split our sample into two groups with high or low-medium alexithymia scores. In conjunction, a cut-off of neuroticism (> = 25), corresponding to the 51% percentile of the theoretical distribution (from 8 to 40), was used to split the sample into high vs. low neuroticism scores. We derived four groups of participants, whose scores were a) high for both alexithymia and neuroticism, b) high only for alexithymia, c) high only for neuroticism, d) low in both constructs. Proportion z tests were used to compare numerosity in each group. Scores at MAIA subscales and at MAIA total scores were compared across the four groups using permutational analysis of variance and post-hoc permutation t-tests with Benjamin-Hochberg correction method to control for type I errors.

## Results

### Preliminary analyses

Preliminary analyses comprised descriptive statistics, comparison of gender and nationality groups, and correlation analyses for the main psychological variables.

Descriptive statistics as a function of participants’ nationality and gender are reported in **S1 Table in S1 File**. For each variable a 2 gender × 2 nationality groups between-subjects’ permutation ANOVA was performed. To control for multiple comparisons (n = 19), the significance threshold was fixed at 0.0026. A significant effect of nationality was found for Consciousness [F(1, 504) = 60.87, p < .001, η^2^p = 0.11] and Openness to experience [F(1, 504) = 47.29, p < .001, η^2^p = 0.09], with US participants scoring higher than Singaporeans in both variables. A significant effect of gender was found for Neuroticism [F(1, 504) = 20.8, p < .001, η^2^p = 0.04], MAIA Attention Regulation [F(1, 504) = 16.0, p < .001, η^2^p = 0.03], and MAIA Not-Worrying [F(1, 504) = 9.38, p = .003, η^2^p = 0.02], with females scoring higher than males in Neuroticism, but lower in the two MAIA subscales. No interaction effects between gender and nationality were found. A significant large effect of nationality [F(1, 504) = 464.14, p < .001, η^2^p = 0.48] and a moderate effect of gender [F(1, 504) = 20.48, p < .001, η^2^p = 0.04] were found for the variable age, with the US group proving older than the Singaporean group and male participants older than female participants. Moreover, after controlling for multiple comparisons, age showed a significant positive correlation with Agreeableness (*r*_*s*_ = .13**), Conscientiousness (*r*_*s*_ = .36***), Openness (*r*_*s*_ = .24***), MAIA total (*r*_*s*_ = .22***), MAIA Not-Worrying (*r*_*s*_ = .16***), MAIA Attention Regulation (*r*_*s*_ = .20***), MAIA Self-Regulation (*r*_*s*_ = .21***), MAIA Body Listening (*r*_*s*_ = .17**), but a negative correlation with Neuroticism (*r*_*s*_ = -.22***).

**S2 Table** in S1 File shows Spearman-moment product correlations between alexithymia, Big Five, and MAIA subdimensions, while **S3 Table** in S1 File shows the same correlations performed on standardized residuals obtained when regressing each variable by gender, nationality, and age. Given the high similarity of the two tables, the following results follow from analyses performed on raw data, without controlling for the effect of demographic variables.

### Network estimation and groups comparisons

**Fig 1:**
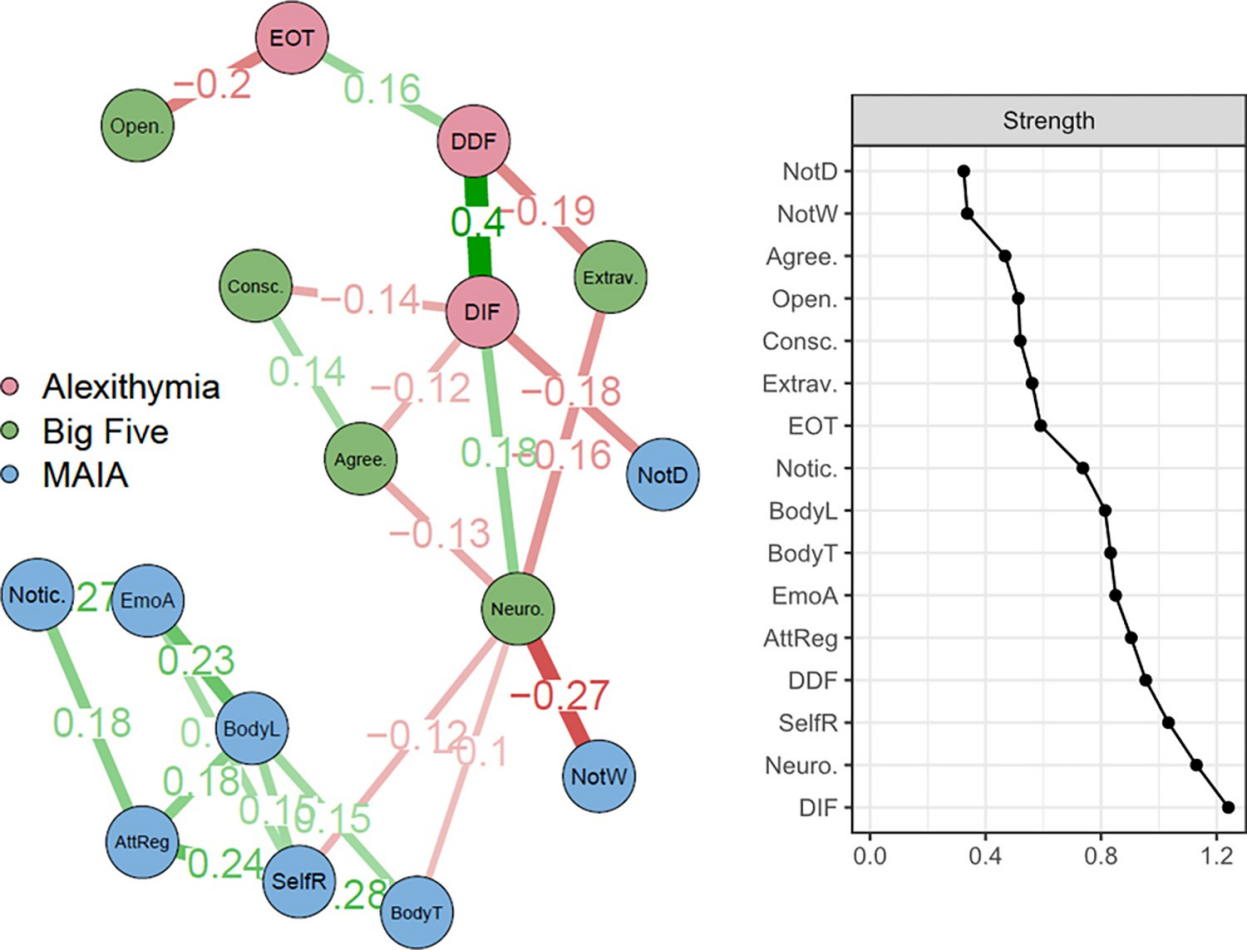
Exploratory partial correlation network for alexithymia, interoception and the Five-Factor Model of personality. Nodes represent TAS-20 subscales (i.e. Difficulty Identifying Feelings [DIF], Difficulty Describing Feelings [DDF], Externally Oriented Thinking [EOT]), Big Five factors (i.e. Neuroticism [Neuro.], Extraversion [Extrav.], Openness [Open.], Agreeableness [Agree.], Conscientiousness [Consc.]), and MAIA subscales (Noticing [Notic.], Not-Distracting [NotD], Not-Worrying [NotW], Attention Regulation [AttReg], Emotional Awareness [EmoA], Self-Regulation [SelfR], Body Listening [BodyL], Body Trusting [BodyT]). Only connections higher than 0.1 are displayed in the figure; the plot for strength centrality metrics is on the right.

**Fig 1** shows the partial Spearman-correlation network built with TAS-20, MAIA and BFI dimensions. As in [76], the majority of MAIA subscores were organized in a strongly connected cluster, with the exception of “Not-Distracting”‘ and “Not-Worrying”. TAS-20 DIF (Difficulty Identifying Feelings) and Neuroticism are the only factors of alexithymia and Big Five to be associated with interoceptive scores. More specifically, Neuroticism shows a negative association with MAIA Not-Worrying (-.27), Self-Regulation (-.12) and Body Trusting (- .10). TAS-20 DIF shows a negative association (- .18) with Not-Distracting. The centrality plot on the right highlights that TAS-20 DIF is the node with the highest strength centrality, followed by Neuroticism and MAIA Self-Regulation.

After applying the cut-off for Alexithymia (A) and Neuroticism (N), we obtained 4 groups: G1) high for both alexithymia and neuroticism (*n* = 93) G2) high only for neuroticism (*n* = 197) G3) high only for alexithymia (*n* = 21) G4) low in both constructs (*n* = 193). Initial proportion tests with post-hoc comparisons showed that the four groups are all different in sample size. The only exception is the comparison of G2 and G4, which does not show significant differences. The group with low neuroticism and high alexithymia (G3) constitutes only 4% of the overall sample. This suggests the low probability of this combination of psychological profiles (high alexithymia and low neuroticism) with respect to all the others.

**Table 1** shows the comparison of interoceptive abilities (MAIA total scores and subscores) for the 4 groups. No significant differences were found for the Noticing and Emotional Awareness subscales. Also, post-hoc analyses for Body Listening failed to show any significant difference between the four groups. Interoceptive abilities as captured by MAIA Not-Worrying and Attention Regulation highlighted the contribution of high Neuroticism, since the group scoring high only in Neuroticism (G2) resulted significantly lower than the group low in both A and N (G4) or the group high only in Alexithymia (G3), while it did not differ from G1, that is the group with high Alexithymia and high Neuroticism. On the contrary, the role of Alexithymia is revealed by the “Not-Distracting” subscale, since the group with high ratings for Alexithymia only (G3) scored significantly lower in “Not-Distracting” than the groups with low Alexithymia and high Neuroticism (G2) and the group low in both constructs (G4), but did not significantly differ from the group high for both Neuroticism and Alexithymia (G1). Finally, G1 presented the lowest values with respect to all the other groups in “Body Trusting” and in MAIA total index, suggesting the interactive role of high A and high N in these factors.

**Table 1:** Mean (standard deviation in parentheses) values of MAIA total scores and subscores for the four groups with Low/High Alexithymia/Neuroticism. F statistics, P value of one-way permutation ANOVAs performed for each variable. P values for post-hoc permutation t-tests are corrected with the Benjamin-Hochberg method.

	M (*SD*)				One-way ANOVA		Post-hoc tests					
	Group 1 High N High A (*n* = 93)	Group 2 High N Low A (*n* = 197)	Group 3 Low N High A (*n* = 21)	Group 4 Low N Low A (*n* = 193)	F	Perm P.	G1-G2	G1-G3	G1-G4	G2-G3	G2-G4	G3-G4
MAIA total	19.87 (4.41)	21.8 (4.22)	23.29 (4.58)	24.48 (4.55)	26.05	< .001	.002	.002	< .001	.146	< .001	.253
MAIA Noticing	3.18 (0.78)	3.25 (0.83)	3.32 (0.81)	3.32 (0.86)	0.691	.563	-	-	-	-	-	-
MAIA Not-Distracting	1.46 (0.80)	2.05 (0.90)	1.54 (0.75)	2.08 (0.95)	12.83	< .001	< .001	.759	< .001	.017	.759	.017
MAIA Not-Worrying	1.98 (0.79)	2.02 (0.83)	2.44 (0.80)	2.75 (0.84)	31.53	< .001	.726	.046	<0.001	.048	< .001	.115
MAIA Attention Reg.	2.72 (0.83)	2.76 (0.74)	3.19 (0.82)	3.2 (0.85)	12.94	< .001	.855	.031	< .001	.021	< .001	.984
MAIA Emo. Awareness	3.13 (0.90)	3.31 (0.87)	3.39 (1.05)	3.35 (0.99)	1.301	.270.	-	-	-	-	-	-
MAIA Self-Regulation	2.42 (1.08)	2.64 (1.00)	2.95 (1.15)	3.34 (0.82)	25.89	< .001	.115	.089	< .001	.199	< .001	.089
MAIA Body Listening	2.32 (1.12)	2.53 (1.09)	2.95 (1.50)	2.69 (1.17)	3.03	.025	.184	.094	.065	.184	.184	.368
MAIA Body Trusting	2.65 (1.25)	3.24 (1.01)	3.51 (0.98)	3.75 (0.90)	25.77	< .001	< .001	.004	< .001	.245	< .001	.245

## Discussion

The aim of this study was to explore the relationship between interoceptive abilities, alexithymia, and the Five-Factor Model of Personality (FFM).

The network model approach allowed us to determine which components of alexithymia and the Five-Factor Model of personality (FFM) are associated with interoception. Because interception is merely a form of perception, this approach allowed us to distinguish components of these two constructs that relate to early perceptual processes from those that reflect higher-order processes.

Difficulty Identifying Feelings (DIF) and Neuroticism were, respectively, the dimensions of alexithymia and FFM most highly associated with interoception. Moreover, they were the components with the highest strength centrality. This means that they played a central role in the network, as bridges between the dimensions of interoception (MAIA subscales) and the other components of alexithymia and FFM. These results are in line with previous research suggesting a strong negative link between self-reported interoceptive abilities and the experience of negative and dysregulated affects, as conceptualized by neuroticism [65, 66] or trait-anxiety [76]. Moreover, these results confirm that it is especially the DIF component of alexithymia that is associated with poor interoceptive abilities [31, 77], mental and somatic health complaints [63], medically unexplained symptoms [62], as well as with the personality trait of neuroticism [60].

Conversely, our analyses show that, when controlling for DIF and Neuroticism, the association between interoception and the other dimensions of alexithymia or of the FFM becomes almost irrelevant (< 0.1). Specifically, the Difficulty Defining Feelings (DDF) component of alexithymia played the role of a connector between DIF and Externally Oriented Thinking (EOT) and also reflected lower scores in Extraversion. Finally, EOT was the component of the alexithymia construct least related to interoceptive measures and was also associated with lower scores in the Openness to Experience dimension of the FFM. These last results help clarify the relationship between openness to experience and alexithymia. While Costa & McCrae [49] suggested that they are close counterparts, this is only true for the EOT dimension of alexithymia; it does not include the role of DIF and DDF. Further, our results support the conclusion that EOT represents the most cognitive component within the construct of alexithymia [25] and that this component, unlike the other two, is insensitive to measures of anxiety or negative affect.

Considering the strong association between the DIF subscale of alexithymia and neuroticism, one might wonder whether the association between alexithymia and interoception can be explained simply by the co-occurrence of neuroticism. This eventuality would represent certainly a major threat to the interoceptive hypothesis of alexithymia [33], which has found strong support in the last years. However, very few studies [78, 79] tested the interoceptive hypothesis while considering co-occurring anxiety, neuroticism, or depression as possible moderating or confounding factors.

To clarify this point, we explored if interoceptive deficits found in people with high alexithymia differ from those in people with high neuroticism and if the co-occurrence of high alexithymia and high neuroticism is associated with general lower interoceptive ability. To do so, we considered how often in our sample high neuroticism and high alexithymia co-occur and how often one condition occurs without the other.

The four groups obtained by combining the conditions of high-low neuroticism and alexithymia (see Table 1) had different numerosity. This already tells us something about the relationship between neuroticism and alexithymia. In fact, among participants with high alexithymia (n = 114), only a small proportion (18%) has alexithymia without neuroticism, while 82% presented both conditions. Contrarily, among participants with high neuroticism (n = 290), 68% presented only high neuroticism, while 32% presented also high alexithymia. From this we can infer that the presence of high alexithymia mostly implies the presence of high neuroticism or similar psychological traits revealing negative affectivity, while the contrary is not necessarily true. This result is in line with the idea that, in alexithymia, the deficit in processing emotions cognitively (low emotional awareness) also results in an inability to down-regulate physiological arousal evoked by stressful or conflicting events [28]. Therefore, we examined if the interoceptive deficits found in the group with highly neurotic traits were the same and were as serious as those in the groups with high alexithymia only, or with both high alexithymia and high neuroticism.

Here we found that the group with high neuroticism only (Group 2) already showed lower interoceptive ability (MAIA total) with respect to the group with both low alexithymia and neuroticism (G4). Specifically, people with a highly neurotic profile only (Group 2) showed higher preoccupation with sensations of pain or discomfort (MAIA Not-Worrying), lower ability to sustain attention to bodily sensations in order to regulate distress (MAIA Attention Reg., MAIA Self-Regulation), and general lack of trust in bodily sensations (Maia Trusting) compared to people low in neuroticism and alexithymia. However, the co-occurrence of high alexithymia and neuroticism (Group 1) further lowers general self-reported interoceptive ability (MAIA total) and, in particular, enhances the maladaptive tendency to ignore sensations of pain or discomfort until they become more severe (MAIA Not-Distracting). This last is also the group with the lowest trust in bodily sensations (MAIA Trusting).

The attention-appraisal model of alexithymia [80] might help us comprehend the specific kinds of interoceptive deficits that appear to be particularly pronounced in alexithymia. Based on Gross’s process model of emotion regulation, which considers four stages for emotion valuation (situation-attention-appraisal-response sequence), Preece et al. [80] suggested that the emotional deficits found in high alexithymia are due to disrupted mechanisms at the stages of attention and appraisal of emotional salient stimuli. Applied to our findings, this model suggests that the reason why the two groups with high alexithymic traits (Group 1, Group 3) paid little attention to sensations of pain or discomfort can be traced back to the tendency of alexithymic subjects to ignore their own emotions. Ultimately, this disrupted attentional mechanism toward internal bodily and emotional signals could be the distinctive feature of people with high alexithymic traits. People with high neuroticism would instead only present a tendency to excessively worry about their bodily sensations, suggesting a disrupted mechanism only at the level of appraisal not attention.

Overall, these findings can be seen as somehow complementary to those of Palser et al. [79], who examined alexithymia in conjunction with self-reported interoception and anxiety. These researchers concluded that, although the correlational nature of their research did not allow them to infer the directionality of the effects, it is feasible that individuals who suffer from anxiety become increasingly alexithymic and sensitive to interoceptive sensations. The self-report nature of our research has the same limitations as Palser et al. [79]. And yet, it offers some support for a different interpretation of the results obtained by these authors. Indeed, Palser and colleagues’ conclusion is not compatible with the idea that alexithymia is a stable personality trait. Thus, their view could apply at most to the case of so-called “secondary alexithymia” [81], which is conceived as a transient condition acquired in adulthood as a consequence of an organic disease, a chronic illness, or an invasive medical treatment (i.e., dialysis, transplant). Our results instead suggest that alexithymia has a high probability (82% in our sample) of co-occuring with behavioral symptoms common to neuroticism and anxiety disorders, as well as the interoceptive deficits manifested in these two conditions. However, the reverse is not necessarily true. This conclusion does not allow us to conclude that alexithymia causes neuroticism. Neuroticism and alexithymia have both been defined as stable personality traits and, as such, it makes little sense to ask which causes the other. Our data show that the psychological profile of alexithymia partially overlaps with that of neuroticism, but they also indicate that high alexithymic traits are mostly accompanied by a high neurotic profile (with the consequent interoceptive deficits), while the reverse is not necessarily true. In fact, one might have high neurotic traits without having high alexithymic traits. In this case, he/she should show less serious overall interoceptive deficits and especially a lower tendency to maladaptively ignore sensations of pain or discomfort.

Of course, our study has some limitations. First, its cross-sectional nature and the use of self-report data prevent us from making strong inferences about the causal direction of one process over the other. Second, the comparison of groups with high neuroticism and/or alexithymia presents some limitations. On the one hand, the number of post-hoc comparisons, as well as the unbalanced sample size of the four groups require caution in the interpretation of results and could provoke an increase in the false positive rate. On the other hand, it is to notice that a clinical cut-off is available for alexithymia, but not for neuroticism. Indeed, the Big Five model represents a dimensional approach to personality. A cut-off to discriminate between individuals with high and low neuroticism was determined by us, using a standard methodology common to many self-report measures. However, this categorical approach is not intended to be applicable in clinical practice. On the contrary, it was proposed for research purposes, mainly to cross-validate findings from network analysis using a different and, thus, complementary analytical approach.

Besides these limitations, our results confirm that atypical interoception represents a common factor underlying decreased socio-emotional competencies in adulthood [11]. Furthermore, our study has theoretical and clinical utility because it highlights the interactive role of neuroticism and alexithymia when defining the type and the severity of interoceptive deficits. Our study suggests that, once neuroticism is detected, it is important to administer a test of alexithymia, in order to better understand the type and severity of the interoceptive deficits manifested by the individual. Finally, these findings suggest that when high neuroticism and alexithymia are detected it might be useful to address interventions and practices (e.g., mindfulness) aimed at enhancing awareness of bodily sensations [82] and, thus, reducing the health risks deriving from disrupted interoception.

## Supporting information

S1 File Detailed information on preliminary analysis, correlation analyses with and without controlling for demographic variables and the partial correlation matrix graphically represented in the network displayed in Fig 1. (DOCX)
