# Supplementary material for: Shared and unique interoceptive deficits in high alexithymia and neuroticism
Source: PLoS One. 2022 Aug 31;17(8):e0273922. doi: 10.1371/journal.pone.0273922 (PMC9432684; doi:10.1371/journal.pone.0273922)
Supplement: S1 File — (DOCX) [file pone.0273922.s001.docx]

# Supplementary Material

**S 1 Table** Descriptive statistics of the sample (N = 504) as a function of nationality and gender. The theoretical range for each psychological variable is as follows: TAS-20 total: 20-100; DIF: 7-35; DDF: 5-25; EOT: 5-40; Neuroticism: 8-40; Extraversion: 8-40; Openness: 10-50; Agreeableness: 9-45; Conscientiousness: 9-45, MAIA total: 8-40; MAIA subscales: 1-5. The sample age range is 18-58.

|  | US Sample (*n* = 257) | | | | | | Singapore Sample (*n* = 247) | | | | | |
| --- | --- | --- | --- | --- | --- | --- | --- | --- | --- | --- | --- | --- |
|  | Females  (*n* = 123) | | Males  (*n* = 134) | | Total  (*n* = 257) | | Females  (*n* = 155) | | Males  (*n* = 92) | | Total  (*n* = 247) | |
|  | M | SD | M | SD | M | SD | M | SD | M | SD | M | SD |
| Age | 28.98 | 5.26 | 29.99 | 4.75 | 29.51 | 5.02 | 21.04 | 1.80 | 23.13 | 1.79 | 21.82 | 2.06 |
| TAS-20 total | 50.32 | 12.13 | 52.37 | 13.01 | 51.39 | 12.62 | 52.77 | 10.04 | 50.20 | 10.19 | 51.81 | 10.15 |
| DIF | 17.35 | 6.09 | 17.87 | 6.92 | 17.62 | 6.53 | 18.59 | 5.04 | 17.45 | 5.32 | 18.17 | 5.17 |
| DDF | 14.31 | 4.67 | 14.98 | 4.56 | 14.66 | 4.62 | 15.42 | 4.27 | 14.28 | 4.01 | 15.00 | 4.20 |
| EOT | 18.66 | 3.94 | 19.52 | 4.12 | 19.11 | 4.05 | 18.76 | 3.61 | 18.47 | 3.67 | 18.65 | 3.63 |
| Neuroticism | 25.85 | 7.29 | 23.38 | 7.52 | 24.56 | 7.50 | 27.68 | 5.78 | 24.59 | 6.21 | 26.53 | 6.12 |
| Extraversion | 22.28 | 7.24 | 22.99 | 7.68 | 22.65 | 7.47 | 21.92 | 5.87 | 23.57 | 6.50 | 22.53 | 6.15 |
| Openness | 36.84 | 6.74 | 37.81 | 6.29 | 37.35 | 6.52 | 33.47 | 5.90 | 33.58 | 5.17 | 33.51 | 5.63 |
| Agreeableness | 33.66 | 6.27 | 33.52 | 6.32 | 33.59 | 6.28 | 32.17 | 5.10 | 32.06 | 5.09 | 32.13 | 5.09 |
| Conscientiousness | 32.81 | 6.46 | 33.09 | 6.66 | 32.96 | 6.56 | 27.93 | 5.20 | 29.53 | 5.37 | 28.53 | 5.31 |
| MAIA total | 22.71 | 5.01 | 23.64 | 5.35 | 23.20 | 5.20 | 21.33 | 4.11 | 22.71 | 3.82 | 21.84 | 4.05 |
| MAIA Noticing | 3.32 | 0.89 | 3.36 | 0.87 | 3.34 | 0.88 | 3.20 | 0.75 | 3.18 | 0.82 | 3.19 | 0.77 |
| MAIA Not-Distracting | 1.95 | 0.95 | 1.92 | 1.02 | 1.93 | 0.99 | 1.91 | 0.89 | 1.94 | 0.82 | 1.92 | 0.86 |
| MAIA Not-Worrying | 2.30 | 0.96 | 2.54 | 0.92 | 2.43 | 0.94 | 2.09 | 0.82 | 2.34 | 0.83 | 2.19 | 0.83 |
| MAIA Attention Reg. | 2.92 | 0.88 | 3.15 | 0.88 | 3.04 | 0.89 | 2.69 | 0.71 | 3.05 | 0.76 | 2.83 | 0.75 |
| MAIA Emo. Awareness | 3.32 | 1.00 | 3.41 | 1.02 | 3.36 | 1.01 | 3.21 | 0.85 | 3.24 | 0.82 | 3.22 | 0.84 |
| MAIA Self-Regulation | 2.89 | 1.01 | 3.04 | 1.06 | 2.97 | 1.04 | 2.68 | 0.99 | 2.98 | 1.01 | 2.79 | 1.01 |
| MAIA Body Listening | 2.65 | 1.19 | 2.74 | 1.24 | 2.70 | 1.22 | 2.42 | 1.03 | 2.48 | 1.15 | 2.44 | 1.07 |
| MAIA Body Trusting | 3.36 | 1.16 | 3.48 | 1.17 | 3.42 | 1.16 | 3.12 | 1.05 | 3.48 | 0.87 | 3.26 | 1.00 |

**S2 Table** Spearman-product correlation matrix. *p < .05, ** p < .01, *** p < .001

|  | 1 | 2 | 3 | 4 | 5 | 6 | 7 | 8 | 9 | 10 | 11 | 12 | 13 | 14 | 15 | 16 | 17 |
| --- | --- | --- | --- | --- | --- | --- | --- | --- | --- | --- | --- | --- | --- | --- | --- | --- | --- |
| 1. TAS-20 total |  |  |  |  |  |  |  |  |  |  |  |  |  |  |  |  |  |
| 2. DIF | 0.87*** |  |  |  |  |  |  |  |  |  |  |  |  |  |  |  |  |
| 3. DDF | 0.85*** | 0.65*** |  |  |  |  |  |  |  |  |  |  |  |  |  |  |  |
| 4. EOT | 0.65*** | 0.34*** | 0.37*** |  |  |  |  |  |  |  |  |  |  |  |  |  |  |
| 5. Neuroticism | 0.44*** | 0.50*** | 0.42*** | 0.08 |  |  |  |  |  |  |  |  |  |  |  |  |  |
| 6. Openness | -0.25*** | -0.12 | -0.17** | -0.33*** | -0.15* |  |  |  |  |  |  |  |  |  |  |  |  |
| 7. Agreeableness | -0.37*** | -0.37*** | -0.30*** | -0.18** | -0.36*** | 0.15* |  |  |  |  |  |  |  |  |  |  |  |
| 8. Conscientiousness | -0.38*** | -0.39*** | -0.31*** | -0.20*** | -0.34*** | 0.16* | 0.33*** |  |  |  |  |  |  |  |  |  |  |
| 9. Extraversion | -0.30*** | -0.20*** | -0.41*** | -0.10 | -0.41*** | 0.24*** | 0.22*** | 0.13 |  |  |  |  |  |  |  |  |  |
| 10. MAIA tot | -0.40*** | -0.36*** | -0.36*** | -0.22*** | -0.44*** | 0.26*** | 0.26*** | 0.31*** | 0.30*** |  |  |  |  |  |  |  |  |
| 11. Noticing | -0.14 | -0.11 | -0.09 | -0.14 | -0.12 | 0.23*** | 0.17** | 0.18** | 0.04 | 0.57*** |  |  |  |  |  |  |  |
| 12. Not-Distracting | -0.31*** | -0.33*** | -0.25*** | -0.15* | -0.13 | -0.10 | 0.10 | 0.14* | 0.01 | 0.19** | -0.16* |  |  |  |  |  |  |
| 13. Not-Worrying | -0.23*** | -0.26*** | -0.20*** | -0.05 | -0.43*** | 0.10 | 0.15* | 0.22*** | 0.19*** | 0.27*** | -0.05 | 0.07 |  |  |  |  |  |
| 14. Attention Reg | -0.22*** | -0.15* | -0.23*** | -0.14 | -0.35*** | 0.26*** | 0.17** | 0.26*** | 0.24*** | 0.74*** | 0.48*** | 0.00 | 0.17** |  |  |  |  |
| 15. Emo. Aware. | -0.17** | -0.10 | -0.14 | -0.15* | -0.09 | 0.24*** | 0.16* | 0.15* | 0.11 | 0.66*** | 0.53*** | -0.08 | -0.07 | 0.48*** |  |  |  |
| 16. Self. Reg. | -0.32*** | -0.31*** | -0.29*** | -0.13 | -0.42*** | 0.18** | 0.21*** | 0.26*** | 0.29*** | 0.80*** | 0.40*** | 0.02 | 0.20*** | 0.59*** | 0.48*** |  |  |
| 17.Body Listen. | -0.20*** | -0.12 | -0.24*** | -0.13 | -0.22*** | 0.19*** | 0.14 | 0.19** | 0.23*** | 0.75*** | 0.43*** | 0.04 | -0.02 | 0.54*** | 0.54*** | 0.54*** |  |
| 18. Body Trust. | -0.39*** | -0.37*** | -0.32*** | -0.21*** | -0.42*** | 0.22*** | 0.22*** | 0.22*** | 0.32*** | 0.73*** | 0.30*** | 0.10 | 0.20*** | 0.46*** | 0.34*** | 0.58*** | 0.47*** |

**S3 Table** Spearman-product correlation matrix. To control for the effect of demographic variables (gender, nationality, age), each psychological variable of interest was regressed by gender, nationality and age and the obtained standardized residuals were correlated *p < .05, ** p < .01, *** p < .001

|  | 1 | 2 | 3 | 4 | 5 | 6 | 7 | 8 | 9 | 10 | 11 | 12 | 13 | 14 | 15 | 16 | 17 |
| --- | --- | --- | --- | --- | --- | --- | --- | --- | --- | --- | --- | --- | --- | --- | --- | --- | --- |
| 1. TAS-20 total |  |  |  |  |  |  |  |  |  |  |  |  |  |  |  |  |  |
| 2. DIF | 0.87*** |  |  |  |  |  |  |  |  |  |  |  |  |  |  |  |  |
| 3. DDF | 0.85*** | 0.65*** |  |  |  |  |  |  |  |  |  |  |  |  |  |  |  |
| 4. EOT | 0.66*** | 0.35*** | 0.39*** |  |  |  |  |  |  |  |  |  |  |  |  |  |  |
| 5. Neuroticism | 0.46*** | 0.51*** | 0.42*** | 0.10 |  |  |  |  |  |  |  |  |  |  |  |  |  |
| 6. Openness | -0.25*** | -0.12 | -0.17** | -0.36*** | -0.10 |  |  |  |  |  |  |  |  |  |  |  |  |
| 7. Agreeableness | -0.37*** | -0.37*** | -0.30*** | -0.20*** | -0.37*** | 0.12 |  |  |  |  |  |  |  |  |  |  |  |
| 8. Conscientiousness | -0.40*** | -0.39*** | -0.33*** | -0.25*** | -0.32*** | 0.07 | 0.32*** |  |  |  |  |  |  |  |  |  |  |
| 9. Extraversion | -0.30*** | -0.20*** | -0.40*** | -0.11 | -0.40*** | 0.24*** | 0.21*** | 0.13 |  |  |  |  |  |  |  |  |  |
| 10. MAIA tot | -0.40*** | -0.36*** | -0.35*** | -0.25*** | -0.41*** | 0.22*** | 0.25*** | 0.27*** | 0.28*** |  |  |  |  |  |  |  |  |
| 11. Noticing | -0.13 | -0.09 | -0.07 | -0.14* | -0.10 | 0.21*** | 0.16* | 0.14 | 0.03 | 0.56*** |  |  |  |  |  |  |  |
| 12. Not-Distracting | -0.31*** | -0.33*** | -0.25*** | -0.15* | -0.14 | -0.10 | 0.10 | 0.16* | 0.01 | 0.19*** | -0.16* |  |  |  |  |  |  |
| 13. Not-Worrying | -0.23*** | -0.25*** | -0.20*** | -0.07 | -0.41*** | 0.06 | 0.14* | 0.17** | 0.18** | 0.25*** | -0.06 | 0.07 |  |  |  |  |  |
| 14. Attention Reg | -0.22*** | -0.15* | -0.21*** | -0.17** | -0.30*** | 0.23*** | 0.15* | 0.21*** | 0.21*** | 0.73*** | 0.48*** | 0.01 | 0.13 |  |  |  |  |
| 15. Emo. Aware. | -0.16* | -0.10 | -0.13 | -0.16* | -0.07 | 0.22*** | 0.15* | 0.12 | 0.09 | 0.66*** | 0.52*** | -0.08 | -0.09 | 0.47*** |  |  |  |
| 16. Self. Reg. | -0.31*** | -0.30*** | -0.27*** | -0.15* | -0.39*** | 0.15* | 0.19*** | 0.23*** | 0.26*** | 0.80*** | 0.39*** | 0.01 | 0.17** | 0.57*** | 0.48*** |  |  |
| 17.Body Listen. | -0.19** | -0.11 | -0.22*** | -0.14* | -0.18** | 0.15* | 0.12 | 0.14* | 0.20*** | 0.74*** | 0.42*** | 0.04 | -0.05 | 0.52*** | 0.53*** | 0.52*** |  |
| 18. Body Trust. | -0.39*** | -0.37*** | -0.31*** | -0.22*** | -0.40*** | 0.18** | 0.21*** | 0.19** | 0.31*** | 0.72*** | 0.28*** | 0.10 | 0.18** | 0.43*** | 0.33*** | 0.57*** | 0.45*** |

**S4 Table** Spearman-product partial correlation matrix graphically represented in the network displayed in Figure 1.

|  | DIF | DDF | EOT | Neuroticism | Openness | Agreeableness | Consciousness | Extraversion | MAIA AttReg | MAIA BodyL | MAIA BodyT | MAIA EmoA | MAIA Notic. | MAIA NotD | MAIA NotW | MAIA SelfR |
| --- | --- | --- | --- | --- | --- | --- | --- | --- | --- | --- | --- | --- | --- | --- | --- | --- |
| DIF | 0 | .4 | .08 | .2 | 0 | -.11 | -.14 | 0 | 0 | 0 | -.11 | 0 | 0 | -.18 | -.02 | 0 |
| DDF | .4 | 0 | .17 | .06 | 0 | -.03 | -.05 | -.18 | 0 | -.01 | 0 | 0 | 0 | -.03 | 0 | -.01 |
| EOT | .08 | .17 | 0 | 0 | -.24 | -.01 | -.06 | 0 | 0 | 0 | -.03 | 0 | 0 | -.01 | 0 | 0 |
| Neuroticism | .2 | .06 | 0 | 0 | 0 | -.14 | -.06 | -.17 | -.03 | 0 | -.09 | 0 | 0 | 0 | -.27 | -.11 |
| Openness | 0 | 0 | -.24 | 0 | 0 | 0 | 0 | .1 | .05 | 0 | 0 | .05 | .04 | -.03 | 0 | 0 |
| Agreeableness | -.11 | -.03 | -.01 | -.14 | 0 | 0 | .14 | .01 | 0 | 0 | 0 | 0 | .03 | 0 | 0 | 0 |
| Conscientiousness | -.14 | -.05 | -.06 | -.06 | 0 | .14 | 0 | 0 | .03 | 0 | 0 | 0 | 0 | 0 | .01 | .03 |
| Extraversion | 0 | -.18 | 0 | -.17 | .1 | .01 | 0 | 0 | 0 | 0 | .09 | 0 | 0 | 0 | 0 | .02 |
| MAIA AttReg | 0 | 0 | 0 | -.03 | .05 | 0 | .03 | 0 | 0 | .17 | .08 | .1 | .2 | 0 | 0 | .24 |
| MAIA BodyL | 0 | -.01 | 0 | 0 | 0 | 0 | 0 | 0 | .17 | 0 | .15 | .24 | .09 | 0 | 0 | .15 |
| MAIA BodyT | -.11 | 0 | -.03 | -.09 | 0 | 0 | 0 | .09 | .08 | .15 | 0 | 0 | 0 | 0 | 0 | .28 |
| MAIA EmoA | 0 | 0 | 0 | 0 | .05 | 0 | 0 | 0 | .1 | .24 | 0 | 0 | .27 | 0 | -.04 | .15 |
| MAIA Notic. | 0 | 0 | 0 | 0 | .04 | .03 | 0 | 0 | .2 | .09 | 0 | .27 | 0 | -.08 | 0 | .04 |
| MAIA NotD | -.18 | -.03 | -.01 | 0 | -.03 | 0 | 0 | 0 | 0 | 0 | 0 | 0 | -.08 | 0 | 0 | 0 |
| MAIA NotW | -.02 | 0 | 0 | -.27 | 0 | 0 | .01 | 0 | 0 | 0 | 0 | -.04 | 0 | 0 | 0 | 0 |
| MAIA SelfR | 0 | -.01 | 0 | -.11 | 0 | 0 | .03 | .02 | .24 | .15 | .28 | .15 | .04 | 0 | 0 | 0 |
